# 

**Published:** 1887-03

**Authors:** 


### TABLE OF CONTENTS.

### Original and Selected Articles.

Epidemic Dysentery, by J. E. Wright, M. D., Columbia, La .......... 89
Some Cases in Ophthalmology, by Dr. W. L. Bullard, Columbus, Ga .......... 97
Ligation of External Iliac Artery for Aneurism of the Femoral, by J. McF. Gaston, M D., Atlanta, Ga .......... 101
Influence of the recent Earthquake shocks in Charleston upon Health, by F. Peyre Porcher, Charleston, S. C .......... 104

### Abstracts and Gleanings.

Health of Los Angeles, Cal,, and Vicinity .......... 108
Arsenic and Antipyrine in Fever .......... 110
Bromide of Ethyl in Conjunction with Chloroform .......... 112
Ice-water Enemata in Treatment of Diarrhœa; Localization of Brain Tumors .......... 115
Union of severed parts; To prevent mammary abscess; Apparent recovery from phthisis .......... 116
Biniodine of mercury in scarlet fever; Acorn Cocoa in infantile diarrhœa; Treatment of ingrowing toe nail; How to syringe the ear .......... 117
Precaution against cholera: Preventive inoculation against phthisis; Ecchinocœa for typhoid .......... 118

### Scientific Items.

The Atmosphere of Caves; Meteor Showers; The Lick Telescope .......... 119
Cementation; Insects as authors of epidemics; What of ghosts .......... 120

### Practical Notes and Formulæ.

Hemorrhoids; Tuberculosis; Fever Mixture .......... 121
"Restorers"; Ointment for grandular affections; To clean tartar from the teeth; Carbolic acid in felo's; Cough mixture .......... 122
Painful dentition; Treatment of the cystitis of Blednorrhagie; Indigestion .......... 123

### Editorials and Miscellaneous.

Editorial Brevities; Commendable; Ogeechee Medical Association .......... 124
Nice prescriptions; How to advertise; Report of the Board of Health of Atlanta, 1886 .......... 125
The Medical Association of Georgia .......... 126
Tributes to Medical Students .......... 127
Books and Pamphlets Received .......... 129
Literary Note from the Century Company .......... 131
Receipts; Special Notes .......... 132

